# Virtual Wound Care in Australian Nursing Homes: Protocol for a Pilot and Feasibility Study

**DOI:** 10.2196/79652

**Published:** 2025-12-02

**Authors:** Heather Russell, Annie Banbury, Katherine Smith, Michelle Barakat-Johnson, Meredith Makeham, Georgina Luscombe

**Affiliations:** 1 School of Rural Health Dubbo/Orange, Faculty of Medicine and Health, The University of Sydney, PO Box 1191, Orange, New South Wales, 2800, Australia, +61253104134; 2 Coviu Global Pty Ltd, Sydney, Australia; 3 The Centre for Health Service Research, Centre for Online Health, The University of Queensland, Brisbane, Australia; 4 Nursing and Midwifery, Sydney Local Health District, Sydney, Australia; 5 Susan Wakil School of Nursing, Faculty of Medicine and Health, The University of Sydney, Sydney, Australia; 6 School of Social Sciences, University of Huddersfield, Huddersfield, United Kingdom; 7 Faculty of Medicine and Health, The University of Sydney, Sydney, Australia

**Keywords:** chronic wound, pilot and feasibility study, nursing homes, homes for the aged, telehealth, virtual care

## Abstract

**Background:**

Chronic wounds, those which have not healed in a timely manner, are a significant health and economic burden. Older people, especially those living in nursing homes, are disproportionately affected by chronic wounds, and effective management and prevention is a persistent challenge. Specialized wound care can improve outcomes; however, access is limited by aged care workforce shortages, fragmented care, and lack of local services, especially in rural and nursing home settings. Virtual wound care interventions such as WoundView (Coviu Global Pty Ltd), a novel computer vision–based artificial intelligence wound analysis app embedded in Coviu’s existing telehealth platform, offer a potential solution to enhance engagement with specialized wound care services.

**Objective:**

This protocol aims to outline a pilot and feasibility study for WoundView to assess the acceptability and feasibility of the intervention in preparation for a planned implementation study. The pilot and feasibility study will estimate recruitment and retention rates along with protocol adherence and adaptations. Qualitative exploration of the acceptability of recruitment processes, training and education, participant assessments, intervention delivery, and secondary outcome measures will inform the development of an implementation study of WoundView.

**Methods:**

The WoundView pilot and feasibility study is a prospective, nonrandomized study in 2 nursing homes in New South Wales, Australia. The research population will comprise up to 10 nursing home residents, 10 to 30 nursing home staff, and 10 wound care clinicians. All resident participants will receive the intervention, WoundView, as routine clinical care throughout the study period. Virtual care will be conducted with a specialized wound care clinic using WoundView’s wound analysis and telehealth features to guide the clinical management of chronic wounds. Wound measures, health-related quality of life, virtual care activity, hospitalization rates, health resource use case studies, and participant satisfaction will be assessed. Nursing home staff and wound care clinicians’ satisfaction with WoundView will be collected through brief surveys and in-depth interviews.

**Results:**

The WoundView pilot and feasibility study was approved by the university’s ethics committee and registered on the Australian New Zealand Clinical Trial Registry. Recruitment and enrollment for the study began in May 2025. Results are expected in the second half of 2025.

**Conclusions:**

The design and implementation of virtual care interventions in nursing homes is an underinvestigated issue. Outcomes from this study will contribute to the design of an implementation study testing WoundView in a range of nursing homes around Australia. The integration of WoundView is expected to transform the use of virtual care for wound management and lead to earlier intervention and increased access to specialist wound advice services for nursing home residents.

## Introduction

### Background

Chronic wounds present a major public health challenge with significant health and economic costs [1]. Chronic wounds are those that have not progressed through the normal stages of healing in a timely and organized process [2] and substantially reduce the quality of life of those affected [3]. The most common types are pressure injuries and chronic lower leg wounds, including diabetic, venous, and arterial ulcers [4]. The global burden of skin and subcutaneous diseases has risen rapidly over the last 15 years, but the lack of international prevalence data for chronic wounds limits the ability to estimate true impact [5]. Based on available figures, an estimated 2.5% of Americans are affected by chronic wounds, the majority of whom are older adults [6]. In Australia, the prevalence of chronic wounds is estimated at 1.9% [7] and accounts for Aus $6.6 billion (US $4.29 billion) per year in costs to health and aged care budgets [8]. Between 2013 and 2017, more than two-thirds of people with chronic wounds who were admitted multiple times to public hospitals in the Australian state of New South Wales (NSW) were aged 65 years or older [9].

Older people, with increasing frailty, impaired skin integrity, and comorbidities such as diabetes, obesity, and peripheral vascular disease, are disproportionately affected by chronic wounds [10,11]. Chronic wounds are particularly common among residents of nursing homes, with an estimated prevalence of pressure injuries of up to 26% [12]. Despite being common, less than 1 in 5 of those with pressure injuries had a wound management record documenting every current injury in a 2018 NSW study of 67 nursing homes [12]. Inadequate wound prevention and treatment was also emphasized by the 2021 Australian Royal Commission into Aged Care Quality and Safety as causing pain, distress, and premature death [13]. Aged care staff giving evidence at the commission reported inadequate knowledge and training in managing pressure injuries effectively [13]. Despite the commission’s recommendation that wound care training and education be improved, inadequate wound care continues to be one of the most common clinical complaints made to the Australian Aged Care Quality and Safety Commission [14].

Evidence-based wound care can improve clinical outcomes of chronic wounds [15]; however, provision of such care in nursing homes can be challenging. Wound care is a specialized field, and in aged care, there is higher variance in wound management and less specialist wound oversight than in other settings [10]. Chronic workforce shortages, high staff turnover, variable wound care expertise among staff, and fragmented and untimely access to expert wound care also limit the provision of consistent and high-quality wound care [13]. Along with evidence-based care, access to clinicians with expertise in wound management can ensure high-quality care and improve resident outcomes; however, in Australia, specialized wound care clinics are generally confined to major centers, which limits in-person attendance by rural and immobile nursing home residents [16]. This geographic centralization of wound care services creates significant barriers to timely assessment and treatment, contributing to delayed wound healing and avoidable complications [17].

Virtual care, health care delivered remotely using information and communication technology services [18], can address access barriers to ensure personalized and timely health care [19] for those unable to attend in-person wound clinics, such as nursing home residents. While the terms virtual care and telehealth are frequently used interchangeably, virtual care is a broader term encompassing telehealth (video or telephone consultations), remote patient monitoring, store-and-forward communications, and website and mobile apps [20]. Virtual care has demonstrated safety and effectiveness in wound healing and can overcome access gaps in chronic wound care [21,22]. Virtual wound care trials have also shown reductions in wound healing times and wound-related hospitalizations [23]; however, there have been few studies undertaken in nursing homes [24]. Furthermore, while current virtual wound care interventions offer wound analysis and measurement capabilities, they lack seamless integration with video telehealth platforms, restricting their effectiveness in remote care.

The number of Australians living in permanent aged care is growing, with 178,000 people aged 65 years or older residing in aged care in 2022, a 3.1% increase since 2017 [25]. Australia has among the highest proportions of older people living in permanent aged care when compared with other similar countries, including the Netherlands, Japan, and Canada [26]. Despite increasing numbers of nursing home residents, there has been a 5.2% drop in aged care providers, attributed to regulatory and financial pressures [27]. To meet the industry’s growing demands and address the imminent implementation of the strengthened Aged Care Quality Standards [28], virtual care innovation, such as artificial intelligence (AI) to assist with wound analysis and remote consultation, is needed to transform the aged care sector, meet the needs of consumers, and improve care outcomes [27]. One such virtual care solution is WoundView, a new AI wound analysis application embedded in an existing telehealth platform, Coviu, which aims to address access barriers to evidence-based and specialized wound care.

### WoundView Application

**Figure 1.**
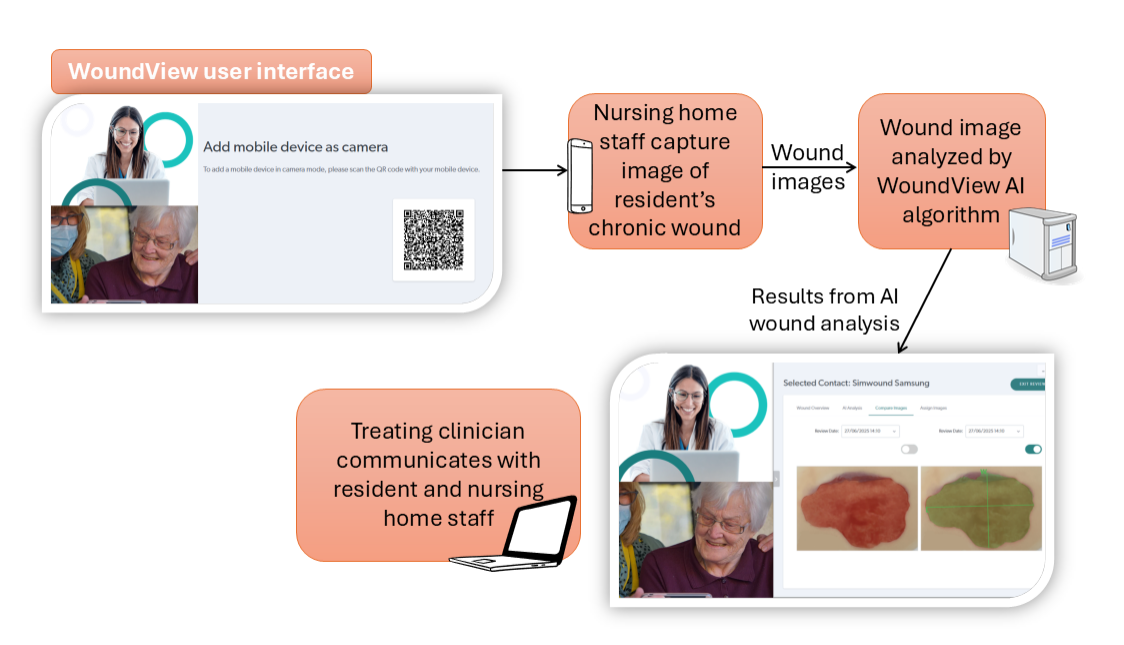
Visualization of WoundView use (reproduced from Coviu Global Pty Ltd [29] and Unsplash [30]). AI: artificial intelligence.

A 5-year, multipartner research project funded by the Australian Government’s Medical Research Future Fund has developed the WoundView application (here referred to as WoundView) to improve access to specialized wound care in nursing homes and other remote settings. WoundView uses a computer vision–based AI algorithm that has been developed and validated using more than a thousand wound images. WoundView produces wound bed segmentation and measurements (ie, length, width, and total area), which can be monitored and compared over time. WoundView (Figure 1) is embedded in the existing telehealth platform, Coviu, allowing for real-time video telehealth between nursing home residents and staff and external wound clinicians. The Coviu platform enables the video call component of the Australian Government–funded Healthdirect Australia, the national virtual public health information service, enabling telehealth use in Australian nursing homes at no cost.

WoundView uses consumer-grade, hand-held devices, including mobile phones, laptops, and tablet devices to assess chronic wounds. The Coviu platform delivers secure, web-based video telehealth. It does not require the download of an app, and all transmitted call data, including images and documents, are transmitted peer-to-peer (ie, between call participants) and protected with end-to-end encryption [31]. This protocol describes a pilot and feasibility study to assess the acceptability and feasibility of WoundView for residents and staff working in nursing homes and external wound care clinicians in NSW, Australia.

### Objectives

The primary objective of the pilot study is to assess the feasibility and acceptability of the proposed approach in preparation for an implementation study on WoundView. Study recruitment and retention and protocol adherence and adaptations will be used to evaluate the primary objective. The secondary objectives are to assess the acceptability of data collection tools for possible inclusion in the implementation study and evaluate collected wound images to verify and update the WoundView AI wound analysis algorithms for future use in nursing homes.

## Methods

### Study Design

The WoundView pilot and feasibility study is a prospective, nonrandomized trial in which all residents with a chronic wound will be eligible to receive the intervention, WoundView. Use of WoundView will be facilitated by staff working at pilot site nursing homes, and wound care will be provided by the Wound Care Command Centre (WCCC) or usual wound care providers. Nursing home staff, WCCC clinicians, and usual wound care providers who use the intervention will also be invited to participate in the study.

The study will determine the acceptability of WoundView and assess the feasibility of selected outcome measures in preparation for an implementation study. The pilot and feasibility study will be reported using the CONSORT (Consolidated Standards of Reporting Trials) extension to pilot and feasibility trials Checklist 1 [32], SPIRIT (Standard Protocol Items: Recommendations for Interventional Trials) statement Checklist 2 and additional guidance on reporting of nonrandomized pilot and feasibility studies by Lancaster and Thabane [33]. A mixed methods approach will be used to assess acceptability and feasibility.

**Figure 2.**
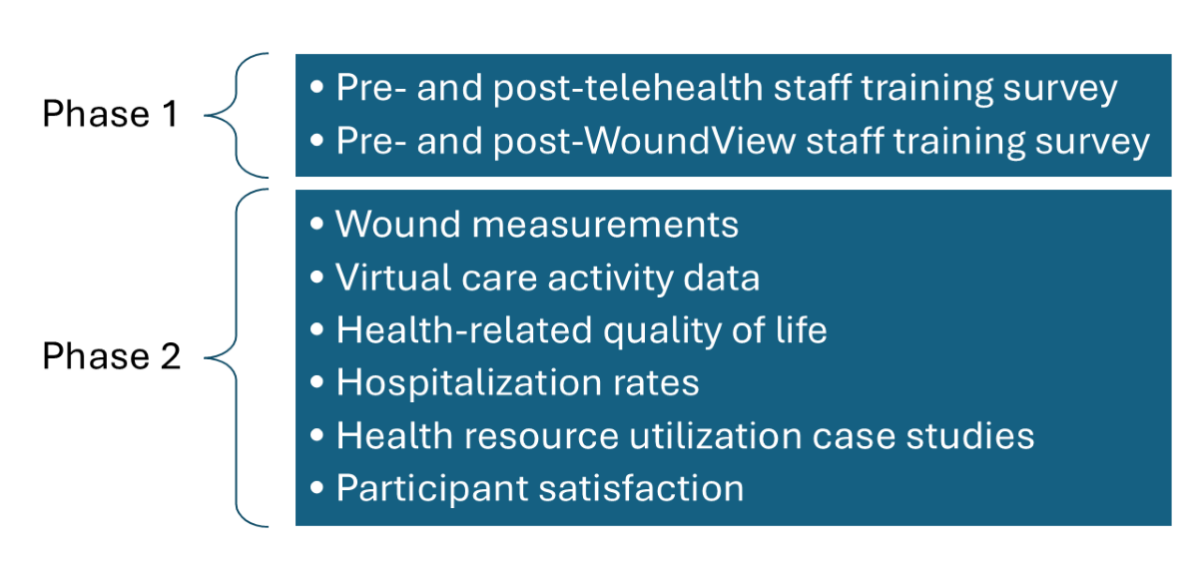
Proposed research plan in 2 phases.

The study will involve 2 phases (Figure 2). During Phase 1, staff working in the pilot site nursing homes will be upskilled in general telehealth and trained to use WoundView. Phase 2 will pilot WoundView and assess the feasibility of the trial design for the planned implementation study.

### Setting

The study will be conducted in 2 private, not-for-profit nursing homes in NSW, Australia. One site is an 80-bed facility in outer regional NSW, as defined by Australian Statistical Geographical Standard Edition 3 [34]. The second site is a 140-bed facility in Sydney, NSW, a major city [34]. The pilot study nursing home sites will be supported by either the WCCC, a team of specialized nurses at the Royal Prince Alfred Virtual Hospital based in Sydney, Australia, or their usual wound care provider.

### Participants

The study will recruit three participant groups: (1) nursing home residents living with chronic wounds, (2) nursing home clinical staff involved in wound care (eg, registered nurses, enrolled nurses, and wound care coordinators), and (3) WCCC clinicians and usual wound care providers, (eg, wound care nurses and general practitioners) who are providing specialized wound care using WoundView.

#### Residents

All residents living in the nursing home pilot sites who have or develop a chronic wound during the study period, regardless of wound type, will be eligible to participate. It is estimated that the number of participants across both sites will be 6 to 10 residents based on wound burden, number of eligible residents, and the relatively brief intervention period of 4 months.

**Figure 3.**
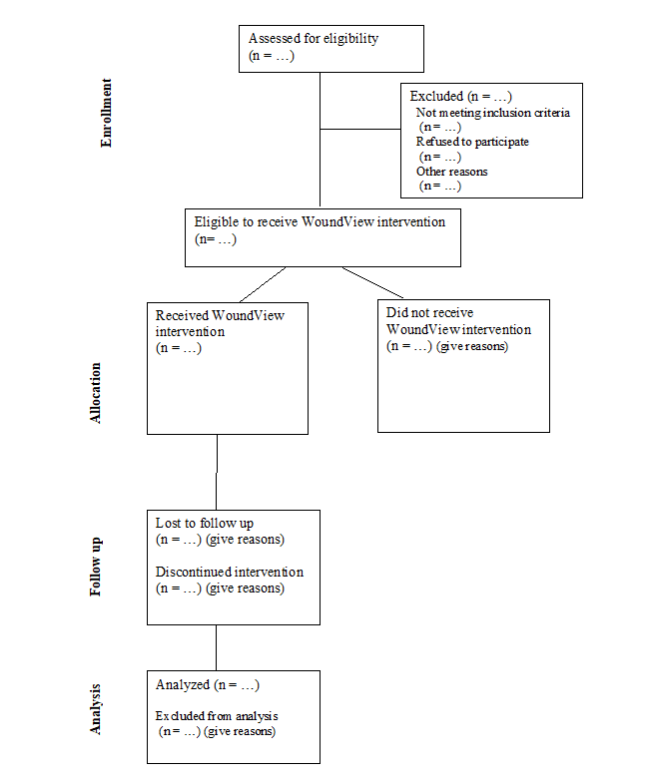
Participant flow diagram.

Recruitment will be undertaken using purposive sampling, where participants who meet the eligibility criteria will be consecutively enrolled into the study. Once enrolled, WoundView will be used to collect and analyze wound images and connect residents to specialized wound management. Resident eligibility criteria are described in Textbox 1 and the participant flow is outlined in Figure 3.

Uncited Textbox 1. **Nursing home resident eligibility criteria.**

**Inclusion criteria**

- Adults aged 18 years or older
- Willing and able to give informed consent for participation in the study
- Have a chronic wound at the start or develop a chronic wound during the study period. A chronic wound is defined as a wound that has been present for 3 or more weeks OR is likely to take 3 or more weeks to heal [35] as determined by nursing home staff and managers with clinical experience in wound care
- Wound must be considered suitable for active treatment as per the advice of the nursing home staff

**Exclusion criteria**

- Cognitive impairment that affects the ability to provide informed consent as determined by the nursing home staff or managers and research team at the point of enrolment
- Sensory, physical, or other health impairment that would preclude data collection participation, as determined by the nursing home staff or managers and research team at the point of enrollment. Other health impairment includes residents who are reliant on medications, for example, participants receiving medication to support end-of-life care or participants with severe mental health conditions where the condition is not adequately controlled by medication at the time of recruitment.
- Have superficial, rapidly healing wounds such as abrasions, blisters, or lacerations.
- Chronic wound located on the genital area
- Not fluent in spoken or written English

Nursing home staff and managers will be asked to identify residents who meet the study eligibility criteria (Textbox 1). Following the identification of potential participants, the research team will approach the resident to explain the study’s nature, purpose, and procedures along with expected duration and potential risks and benefits. Potential participants will be provided with a research information sheet and consent form. Residents will be invited to discuss their potential participation with support people such as next of kin, family, friends, or their enduring guardian. Following a 3-day cooling-off period, the research team will contact the resident to ascertain their preference to be involved in the study. To avoid perceived or actual coercion, recruitment and consent will be undertaken by the research team, who are independent of the nursing home.

Nursing home residents who verbally consent to participate will be invited to provide written consent at the beginning of the study. Throughout the study, WoundView will be used as routine clinical care, and further consent will not be sought for each episode of use of WoundView in wound management. At the conclusion of the study, residents will be invited to reconfirm their consent verbally, prior to the final data collection.

Resident participants may withdraw from the research study at any time on request, with assurance that their withdrawal will not affect their relationship with their nursing home or the university. To avoid perceived or actual coercion, written consent of nursing home residents will be undertaken by the research team, who are independent of the nursing home. Participants will not receive any payment or reimbursement for their participation.

#### Nursing Home and WCCC Clinicians

All clinical staff working at the participating nursing homes, WCCC clinicians, and usual wound care providers are eligible to participate if they are aged 18 years or older and can undertake data collection in English.

During Phase 1, telehealth and WoundView education and training will be conducted with nursing home staff nominated by their organization to participate. Nursing home staff will be provided with access to a curated package of self-directed online telehealth videos [36], which will take approximately 20 to 30 minutes to complete. Following telehealth training, face-to-face WoundView training will be conducted by the research team, lasting 30 to 45 minutes. Training will include how to set up and conduct a WoundView call and optimal wound image capture, such as the use of direct camera angle and adequate lighting to minimize shadow. Nursing home sites will also receive a recording of the training session, quick reference documents, and a site-specific flowchart to integrate WoundView into usual care. A brief training session, including a practice call, will be undertaken with the WCCC staff prior to commencement of the study.

Nursing home staff who participate in training will be invited to undertake a brief, anonymous online evaluation prior to and after the training sessions. The trainer will describe the survey, display a QR code for the survey, and leave the room to allow participants to decide whether to complete the survey. The number of nursing home staff participants is expected to be 10 to 30.

In Phase 2, nursing home staff, WCCC clinicians, and usual wound care providers will be invited to participate in a brief online survey and an in-depth, semistructured interview with a member of the research team lasting approximately 20 to 30 minutes. Nursing home management will be asked to assist by making staff aware of the study through email correspondence or posting advertisements in appropriate locations, such as staffrooms. WCCC clinicians and usual wound care providers will be invited to participate through email correspondence. The survey will be accessible online along with the Participant Information Statement and consent form. Those interested in being interviewed will be able to self-identify at the end of the survey or initiate contact with the research team to arrange an interview. The number of survey participants is expected to be 5 to 10, and interview participants are estimated to be 5.

Participants will not receive any payment or reimbursement for their participation. Participants may withdraw from the research study at any time with assurance that their withdrawal will not affect their relationship with their employer or the university. To avoid perceived or actual coercion, written consent of nursing home staff, WCCC clinicians, and usual wound care providers will be undertaken by the research team, who are independent of the nursing home.

### Intervention

The intervention, WoundView, is a novel computer vision–based AI wound analysis application integrated into the Coviu telehealth platform. WoundView is a software component that extends the functionality of the Coviu platform, without requiring changes to its core code. WoundView will link residents with chronic wounds and their clinical care team to specialized wound care clinicians at the WCCC in Sydney, Australia, or their usual wound care providers, who will deliver expert wound care based on clinical data gathered during video telehealth, AI wound analysis, including wound measurements and segmentation, and resident and family preferences.

WoundView requires an initial referral from the participating nursing home to the WCCC or usual care provider for an eligible resident. An appointment is scheduled using the Coviu platform, and the resident, nursing home staff, and wound care clinician attend at the scheduled time. At each use of WoundView, wound image and video data will be gathered by nursing home staff who will take a series of wound photographs prior to or during the WoundView call and submit these for AI wound analysis. The inclusion of an ArUco marker (a marker of known size) in each wound image enables AI wound size analysis using the WoundView AI algorithm. The AI wound analysis provides wound measures such as length, width, and area, along with wound segmentation. Live video stream of the wound will also be available during the call, providing treating clinicians with detailed, close-up vision of the wound, an innovative feature of WoundView. The wound care clinician provides clinical advice about wound management, including cleaning, debridement, dressings, and frequency of dressing change. Further wound review is arranged as advised by the wound care clinician.

### Outcomes

The primary outcome will be a narrative description of study feasibility, including participant recruitment and retention, protocol adherence and adaptations, and stakeholder acceptability (Table 1). A traffic light system will guide progression to an implementation study [37], red (stop, main study not feasible), amber (feasible with modifications or close monitoring), and green (continue without modifications) [38]. A recruitment rate of greater than 50% (defined as the percentage of eligible residents recruited) and retention of greater than 75% (proportion of participants who remain in the pilot for its duration) will be considered acceptable to proceed with the implementation study without modification. Protocol adherence, the degree to which the WoundView intervention and research protocol were implemented as intended, greater than 75% will be considered acceptable. Additionally, regular stakeholder input and agreement on the interpretation of pilot findings will be sought [37]. Acceptability will be assessed by measuring participants’ satisfaction with study assessment instruments, and the use, delivery, barriers, and enablers to the intervention, WoundView. Data collection instruments are described in the section below. Secondary outcomes, including wound measurements, virtual care activity, health-related quality of life (HRQoL), hospitalization rates, health resource use case studies, and patient and staff satisfaction, will be assessed for feasibility of inclusion in a planned implementation study.

**Table 1.** Overview of outcomes and methods.

Outcome levels	Methods and approaches
Primary	
Feasibility	
Recruitment	Percentage of eligible residents recruited
Retention	Proportion of participants who remain in the pilot for its duration
Adherence	Degree to which the WoundView intervention and research protocol were implemented as intended (eg, staff training logs and surveys), WoundView use and activity, resident, staff, and clinician-reported WoundView use
Satisfaction	Pre- and posttraining staff surveys Staff satisfaction survey Short assessment of patient satisfaction In-depth semistructured interviews (residents, nursing home staff, and wound care clinicians) exploring WoundView acceptability
Secondary	
User confidence and skills	Pre- and posttraining staff surveys
Time to 35% reduction in wound area Time to complete healing Percentage area reduction Gross area reduction	Wound measurements (eg, width, length, and area)
WoundView use	Virtual care activity (eg, call participants, guest, or host), length of call (call, time and date), and AI wound measurements (eg, length, width, and calculated wound area)
Quality of life	Wound-QoL-14^a^ EQ-5D-5L^b^
Care costs	Hospitalization rates Emergency department attendance rates Health resource use case studies
Adverse events	Wound recurrence Adverse events (eg, death, amputation, infection, hospitalization, ED^c^ attendance, and allergic reactions).

a Wound-QoL-14: Wound Quality of Life-14.

b EQ-5D-5L: EuroQoL-5 Dimensions-5 Levels.

c ED: emergency department.

### Data Collection and Analysis

#### Phase 1: Pre- and Posttraining Evaluation

Nursing home staff participating in telehealth and WoundView education and training will be invited to rate their pre- and posttraining confidence and skills, along with their satisfaction with the training in a brief, anonymous online survey.

#### Phase 2: Pilot and Feasibility Study

Phase 2 of the pilot and feasibility study will comprise several components with data collection timepoints at baseline, during the intervention, and at the end of the study. Study outcomes, methods, and data analysis are outlined below.

##### Feasibility

Recruitment, defined as the percentage of eligible residents recruited, retention, the proportion of participants who remain in the pilot for its duration, and protocol adherence, the degree to which the WoundView intervention and research protocol were implemented as intended, will be assessed. Acceptability will be assessed by measuring participants’ satisfaction with study assessment instruments, and the use, delivery, barriers, and enablers to the intervention using staff training and satisfaction surveys, patient satisfaction survey, and in-depth semistructured interviews with residents, nursing home staff, and wound care clinicians.

##### Wound Measurements

At baseline, the research team will record a measurement of the widest dimension of the wound or wounds (length) and the second widest dimension perpendicular to the first widest dimension (width) using a single-use paper tape measure. The wound type, duration, and location will be recorded on the data collection form. For each wound, a series of still photos will be captured using WoundView. The wound images will be assessed using WoundView’s AI analysis to determine wound width, length, and total wound area and compared to the manual wound measurements.

At subsequent WoundView use, AI wound data will be used to calculate time to 35% reduction in wound area and time to complete healing, defined as full re-epithelialization or closure [39]. Percentage area reduction and gross area reduction will be calculated from raw data at various intervals (eg, 1 week). If a resident participant withdraws from the pilot, their most recent wound measurements will be analyzed as the final data point.

##### Virtual Care Activity Data

During the study period, data on all episodes of WoundView use will be extracted from the Coviu platform. Virtual care activity data generated will include call participants (eg, guest or host), length of call, call time and date, and AI wound measurements (length, width, and calculated wound area).

##### HRQoL

HRQoL will be collected using wound-specific (Wound Quality of Life-14 [Wound-QoL-14]) and general (EuroQoL-5 Dimensions-5 Levels [EQ-5D-5L]) questionnaires at baseline and at the conclusion of the pilot study.

##### Wound-QoL-14

Wound-QoL-14, a shortened version of the Wound-QoL-17, is a disease-specific, HRQoL measure for patients with chronic wounds [40]. The Wound-QoL-14 asks respondents to rate impairment over the last 7 days in the subscales body (pain, odor, discharge, and sleep), psyche (unhappy, frustrated, worried, and fear of worsening), and everyday life (moving about, everyday activities, leisure activities, activities with others, and depending on help) [41]. A global Wound-QoL-14 score can be calculated if at least 75% of the 14 items have been answered [42]. Wound-QoL-14 has demonstrated high internal consistency (Cronbach α= 0.779‐0.925), moderate to good test-retest reliability (intraclass correlation coefficient: 0.618‐0.808), and convergent validity showing highest correlations with global HRQoL rating (*r*=0.751) and Dermatology Life Quality Index (DLQI) rating (*r*=0.681) [43]. Wound-QoL-14 has been shown to be valid to assess HRQoL of patients with chronic wounds, acceptable for use in cross-cultural settings [41] and a practical option in time-poor settings [43].

##### EQ-5D-5L

EQ-5D-5L is a general HRQoL measure which consists of the EQ-5D descriptive system and the EQ visual analog scale [44]. The EQ-5D descriptive system comprises 5 dimensions (mobility, self-care, usual activities, pain and discomfort, and anxiety and depression), each measured at 5 levels (no problems, slight problems, moderate problems, severe problems, and extreme problems) describing the respondent’s health state [45]. The EQ visual analog scale records the patient’s self-rated health on a vertical visual analog scale where the endpoints are labeled ‘the best health you can imagine’ and ‘the worst health you can imagine’ and can be used as a quantitative measure of health outcome that reflects the patient’s own judgment [45]. The EQ-5D-5L shows excellent psychometric properties across a range of populations, health conditions, and settings [46].

##### Hospitalization Rates

At the conclusion of the intervention period, an audit of the electronic medical records (EMRs) of participating residents will be undertaken to capture the frequency and details of transfers to emergency departments (EDs) and hospital admissions for wound-related care. The timeframe of interest will be 12 months prior to the chart audit. The EMR will be audited to capture relevant text entries and discharge summaries associated with wound-related ED visits and hospital admissions.

##### Health Resource Utilization Case Studies

Health resource utilization associated with the management of chronic wounds in nursing homes will be estimated through a series of wound care episode case studies. An EMR audit and observation of usual wound care will be undertaken with a sample of 3 to 4 consenting residents. The research team will observe the wound dressing change, document the labor and consumables required for wound management, and undertake a chart audit to estimate the number of wound management episodes during the study period. Where possible, more than 1 dressing change for the same resident will be observed, and the total labor and consumables will be averaged.

##### Adverse Events

An audit of participating residents’ EMRs will be undertaken to capture rates of wound recurrence and adverse events (AEs; eg, death, amputation, infection, hospitalization, ED attendance, and allergic reactions) at the completion of the intervention period.

##### Participant Satisfaction

###### Nursing Home Residents

Nursing home resident satisfaction will be assessed using a validated instrument, the Short Assessment Patient Satisfaction (SAPS) and through in-depth semistructured interviews with the research team.

###### SAPS

The SAPS is a brief, validated questionnaire used to assess patient satisfaction across 7 domains, including treatment satisfaction, explanation of treatment results, clinician care, participation in medical decision-making, respect by clinician, time with clinician, and satisfaction with care [47]. The SAPS’ internal psychometric properties exceed standard psychometric standards (Cronbach α=0.86), and it discriminates at least as well as other longer patient satisfaction measures [48]. SAPS will be administered at the conclusion of the pilot to measure participants’ satisfaction with their experience of care in the study.

###### In-depth Semistructured Interviews

All participating nursing home residents will also be invited to take part in an in-depth, semistructured interview conducted by the research team. Interviews will be undertaken in person in a private room or via Microsoft Teams and last for approximately 20 to 30 minutes. The interviews will explore the acceptability of the recruitment processes, assessments, and intervention delivery. Virtual interviews will be digitally recorded in Microsoft Teams and transcribed using Microsoft Teams or Word. In-person interviews will be recorded using a hand-held digital recorder and transcribed in Microsoft Word by the first author. Participants will be provided with the choice to review a transcribed copy of the interview by checking the appropriate box on the consent form.

Inductive thematic analysis of interview data will be guided by the feasibility objectives of the pilot (Table 1). Qualitative data will be organized and thematically analyzed using NVIVO 15 software (Lumivero). Following familiarization with the data, line-by-line coding will be undertaken to identify initial codes [49]. The research team, consisting of 2 to 3 independent researchers, will undertake reflexive journaling during the coding process to consider the emerging themes [50]. To create a robust codebook, researchers will meet regularly to review candidate themes for distinction and coherence [51]. After a consensus is reached, the codebook will include definitions and examples of each code, and the coding process will continue until data saturation (ie, no new themes are observed) [52]. Thematic analysis will be conducted in accordance with the Reflexive Thematic Analysis Reporting Guidelines [53]. A sample of at least 5 interviews will be undertaken with each target group: nursing home residents, nursing home staff, and wound care clinicians.

###### Nursing Home and WCCC Clinicians

All nursing home staff, WCCC clinicians, and usual wound care providers who use WoundView during the study period at pilot sites will be invited to rate their satisfaction in a brief anonymous online survey adapted from Barakat-Johnson et al [54] All nursing home staff, WCCC clinicians, and usual wound care providers involved in the study will also be invited to participate in 20 to 30 minute, in-depth, semistructured interviews conducted by the research team. Interviews will be undertaken in person in a private room or via Microsoft Teams for the resident interviews and will explore recruitment, training and education, assessment tools, and the delivery of the intervention. See In-depth semistructured interviews for details on coding and analysis.

### Statistical Analysis

ED visits and hospital admission data, including length of stay, reason for admission, diagnosis on discharge, and discharge location, will be recorded and analyzed using descriptive statistics. Descriptive statistics will also be used to illustrate the change in wound size across the intervention period as well as to summarize HRQoL, AEs, and satisfaction survey results. All analyses will be intention-to-treat. An estimate of costs will be derived from the total number of dressing changes and extrapolated as per Wilson et al [55]. The actual and projected treatment costs will be calculated and extrapolated to estimate the overall cost of wound care in the pilot nursing home sites. Estimated costs will be compared with other Australian estimates in nursing home settings [56].

### Verification and Update of WoundView AI Wound Analysis Algorithm

Wound images collected during the WoundView pilot and feasibility study will be used as part of the verification and update of the WoundView AI wound analysis algorithm. To verify the quality of the AI wound analysis algorithm, a ground truth mask and an AI-predicted mask will be created and compared for each wound image. Masks are pictorial representations that isolate a specific part of an image, such as the wound bed from the background. To create the ground truth mask used to verify the performance of the AI wound analysis algorithm, wound images collected using WoundView during the pilot will be manually annotated by experienced wound care clinicians using the Computer Vision Annotation Tool (Intel Corporation), a free, open-source, web-based image and video annotation tool. Random checks of the annotation accuracy will be conducted by 3 wound care experts. To verify the performance of the AI model, ground truth annotations will be compared with the AI-predicted mask. Multimedia Appendix 1 outlines data descriptions, study devices, data handling, and storage processes, as well as data retention for the study. Explicit consent will be sought from participating residents for the use of captured wound images in the verification and update of the WoundView AI wound analysis algorithm.

### Monitoring

Conduct of the pilot will be overseen by a data monitoring group consisting of members from the research team from the university and Coviu. The Chief Investigator is a member of the data monitoring group and will make the final decision on ceasing or proceeding with the trial. The group will meet regularly to discuss pilot data quality and completeness. The group will review source documents (eg, wound assessments, virtual health activity, health resource use case studies, and HRQoL measures) along with compliance with informed consent and eligibility criteria and monitoring of AEs and endpoints. Additionally, data storage and handling practices, particularly with respect to the transfer of wound images (Multimedia Appendix 1), will be regularly reviewed. If a problem is identified (eg, missing study documents), the team will work to resolve the issue. All parties involved will keep participants’ data strictly confidential.

### Ethical Considerations

The trial has been registered with the Australian and New Zealand Clinical Trials Registry on June 2, 2025 (ACTRN12625000565448). Ethical approval was received from the University of Sydney Human Research Ethics Committee (HREC; 2024/HE001356). Informed written consent will be obtained from all participants. Participants will not receive any payment or reimbursement for their participation. Participants may withdraw from the research study at any time with assurance that their withdrawal will not affect their relationship with the nursing home, employer, or the university. The pilot and feasibility study will collect personal information, which will be reidentifiable and securely stored in HIPAA (Health Insurance Portability and Accountability Act)–compliant REDCap (Research Electronic Data Capture; Vanderbilt University), a secure web application for managing online surveys and databases hosted by the University of Sydney in NSW, Australia. Only approved members of the research team will have access to personal information. All information will be subject to the University’s HREC confidentiality policies. Figure 1 uses 2 images of people. These are generic images and are not of research participants. The image of the clinician using the laptop was obtained with permission from Coviu Global Pty Ltd (developer of WoundView) [29]. The image of the older woman and nurse using a mobile device was sourced from Unsplash [30], which grants “irrevocable, nonexclusive, worldwide copyright license to download, copy, modify, distribute, perform, and use images from Unsplash for free, including for commercial purposes, without permission from or attributing the photographer or Unsplash.”

### Protocol Amendments

Any required changes to the protocol will be submitted as amendments to the University of Sydney’s HREC for approval before implementation. In an emergency, deviations from the protocol may be made to protect human subjects’ rights, safety, or well-being without the HREC’s prior approval. Such deviations will be promptly documented and reported to the HREC.

### Dissemination

Results of the study will be included in reports to stakeholders, journal publications, and conference presentations as part of the dissemination of the broader study’s outcomes. Findings will be provided in such a way that participants cannot be identified.

## Results

**Figure 4.**
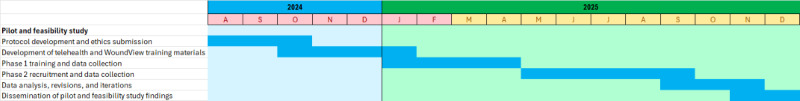
WoundView pilot and feasibility study timeline.

Approval for the pilot and feasibility study was granted by the University of Sydney HREC in October 2024 (2024/HREC1356), and the study has been registered with the Australian and New Zealand Clinical Trials Registry. Preliminary work with the 2 pilot nursing homes commenced in January 2025, and telehealth and WoundView training began in March 2025. The first study participants were recruited in May 2025. A timeline of proposed research activities is outlined in Figure 4. Results from the study will be analyzed and presented in tabular, graphical, and narrative formats.

## Discussion

### Anticipated Findings

Chronic wounds are common among nursing home residents, detrimentally affecting their quality of life and contributing to increased risk of premature death. When compared with other populations, nursing home residents experience significant barriers accessing timely and high-quality wound care. While research in virtual wound care interventions is rapidly expanding, little is known about its use in nursing homes despite the high burden of disease and substantial impact on health outcomes.

The development of a novel computer vision–based AI wound analysis application embedded in an existing telehealth platform is warranted in offering clinicians and residents a “one-stop shop.” It is expected that the findings of this study will provide sufficient methodological evidence regarding the design and planning of the intervention to warrant an implementation study. The methodological evidence will include an approach to participant recruitment, retention, and adherence to the intervention. Although this pilot and feasibility study is not powered to detect statistically significant effects, the findings will provide insights to optimize the design of a larger scale implementation study and strengthen the research approach. Results will also inform the implementation and evaluation of new virtual wound care interventions in nursing homes.

### Conclusions

The pilot study will assess the acceptability and feasibility of a novel wound care telehealth intervention, WoundView, and will contribute to the design of an implementation study testing WoundView in a range of nursing homes in Australia. The integration of WoundView is expected to transform the use of telehealth for wound management and lead to earlier intervention and better access to specialist wound care services for nursing home residents.

## Supplementary material

10.2196/79652 Multimedia Appendix 1 WoundView pilot and feasibility study data flows and retention.

10.2196/79652 Checklist 1 CONSORT checklist.

10.2196/79652 Checklist 2 SPIRIT 2025 checklist.

## Abbreviations

AE: adverse event
AI: artificial intelligence
CONSORT: Consolidated Standards of Reporting Trials
ED: emergency department
EMR: electronic medical record
EQ-5D-5L: EuroQoL-5 Dimensions-5 Levels
HREC: Human Research Ethics Committee
HRQoL: health-related quality of life
NSW: New South Wales
SAPS: Short Assessment of Patient Satisfaction
SPIRIT: Standard Protocol Items: Recommendations for Interventional Trials
WCCC: Wound Care Command Centre
Wound-QoL-14: Wound Quality of Life-14
