# Supplementary material for: Virtual Wound Care in Australian Nursing Homes: Protocol for a Pilot and Feasibility Study
Source: JMIR Res Protoc. 2025 Dec 2;14:e79652. doi: 10.2196/79652 (PMC12671902; doi:10.2196/79652)
Supplement: Multimedia Appendix 1 [file resprot-v14-e79652-s001.docx]

**Table S1.** WoundView pilot and feasibility study data flows, management and retention.

| **Data description** | Two categories of data will be collection in the WoundView pilot and feasibility study:   1. Highly protected    - De-identified demographic and clinical data    - De-identified wound images and videos    - De-identified audio recordings and transcriptions of in-depth interviews    - Paper and electronic participant consent forms (PCFs) 2. Protected    - Anonymous evaluation of training    - Anonymous satisfaction survey responses   Confidentiality of participating residents will be maintained through the de-identification of all research data as soon as practicable after data linkage between resident name and unique identifier has occurred. Resident’s names will be used to link data and a unique identifier will be used after the data is linked. Earlier de-identification of resident data is not deemed practical because nursing home staff are often away from the computer and may not be able to easily access unique identifiers.  Master key linking participants to their unique identifier will be stored in a separate folder on the University of Sydney Research Data Store (RDS, a secure server suitable for storage of highly protected data). Only the Chief Investigator and Nurse Researcher /PhD student will have access to identifiable information such as consent forms and the master key linking participants to their unique identifiers. Paper forms (PCF and data collection forms) will be stored in a locked office at the School of Rural Health accessible to University study investigators only.  Anonymous nursing home staff and wound care clinician survey results from Phases 1 and 2 will be recorded directly in REDCap. Audio recordings and de-identified transcriptions of in-depth interviews with residents, nursing home staff, and wound care clinicians will be saved in separate sub-folders within the Research Data Store (RDS). |
| --- | --- |
| **Study devices** – *access control and security* | Study laptop – The University of Sydney will provision a dedicated laptop to be used for prospective data collection authenticated using personal login credentials. Encrypted at rest using Bitlocker (AES-XTS 256). |
| **Storage platforms** | 1. **Commonwealth Scientific and Industrial Research Organisation (CSIRO) server** (managed by CSIRO Data 61): CSIRO provisions a dedicated workstation for the WoundView project with its own computer, storage, and user access control and auditing. This server is configured to support encryption at rest, using the Linux Unified Key Setup (LUKS) using Bitlocker (AES-XTS 256-bit) or similar software/encryption scheme. Approved CSIRO-affiliated researchers will access this workstation using the network communication protocol, secure shell protocol (SSH) which will provide a secure, encrypted communication between the researcher and the workstation. 2. **Amazon Web Services (AWS) S3 buckets** (managed by Coviu): Secure, HIPAAA compliant internet data storage service with regional restrictions which enables all study data to be stored in Australia. AWS encrypts all data during transmission and at rest and is equivalent to the standards set by the University of Sydney’s policies for the secure collection and storage of research data. An object store on AWS S3 will be arranged by Coviu, the lead organisation on the MRFF grant funding this research. Coviu will grant AWS access to study investigators as required. 3. **Computer Vision Annotation Tool (CVAT)** deployed on AWS EC2 (managed by Coviu): CVAT will be deployed on an AWS EC2 instance on AWS is which HIPAAA compliant internet data storage equivalent to the standards set by University of Sydney policies for the secure collection and storage of research data. Wound annotators will have access restricted to CVAT only for the purposes of annotation. 4. **Research Data Store** **(RDS)** (managed by The University of Sydney): A University supported platform for the management of protected/highly protected data. The RDS is a secure, enterprise-grade Network Attached Storage device located within New South Wales (NSW), Australia. 5. **REDCap** (managed by The University of Sydney): an online data capture tool that stores data on secure servers within NSW, Australia. Access will be restricted to University investigators. All demographic and clinical data will be stored on the University’s licensed instance of REDCap. 6. **OneDrive** (managed by The University of Sydney): will be used to share data with CSIRO. Note: there will be no identifiable data shared via OneDrive. Access to the OneDrive folder will be restricted to CSIRO/CSIRO affiliated staff and the University research team members as required 7. **WoundView** (managed by Coviu Pty Ltd): wound images and video data acquired using WoundView will be restricted to the study investigators as required via a unique login and password. De-identified images/video will be transmitted to the Coviu AWS S3 bucket and are encrypted in transit and at rest. Data captured in the WoundView plugin transmitted to the Coviu AWS S3 bucket will be downloaded to the University of Sydney RDS. |
| **Data handling and storage** | 1. Demographic and clinical data is collected by the Nurse Researchers/PhD student on the University dedicated study laptop and entered directly into REDCap. 2. Wound and telehealth consult/assessment data (including wound images) from WoundView will be accessible from the Coviu Telehealth Platform to University investigators via AWS S3 for upload to RDS. **Note**: Data captured via the WoundView plugin will be transmitted wirelessly to the Coviu AWS S3 bucket. Data will be downloaded by the University Nurse Researchers/PhD student from AWS to the University RDS. 3. Wound images captured in WoundView will be sent to CVAT for annotation. 4. Annotation will be downloaded from CVAT to the RDS for ground truth masks to be generated using Python script. 5. CSIRO will download images from Coviu AWS S3 bucket to create predicted wound masks using the AWS application programming interface (API) credentials provided by Coviu. The download will be encrypted in transit using hypertext protocol secure (HTTPS) and transport layer security (TLS). 6. Deidentified predicted wound masks are generated and uploaded to the University OneDrive. 7. Evaluation script compares ground truth mask against predicted mask. 8. Survey responses from Phases 1 and 2 will be entered directly into REDCap by participants and saved on the RDS. 9. Audio recordings and de-identified transcriptions of in-depth interviews with residents, nursing home staff, and wound care clinicians will be saved in separate sub-folders within the RDS. 10. Master key linking participants to their unique study number will be stored in a separate folder on the RDS. 11. At the end of the study, all project data will be copied from the RDS to AWS S3 for long-term storage. |
| **Data retention** | - Wound image and video data collected via WoundView will be retained and stored on Amazon Web Service (AWS) S3 by Coviu for five years from when the product is no longer commercially available, as per the Australian Regulatory Guidelines for Medical Devices. - Data stored on the University of Sydney Research Data Store (RDS) will be retained for a minimum of five years as per the National Health and Medical Research Council (NHMRC) guidelines. - Data stored on the Commonwealth Scientific and Industrial Research Organisation (CSIRO) Server will be deleted at the completion of the entire study. - At the completion of the retention period, all data will be permanently deleted from the RDS and AWS S3. Papers forms will be disposed of via a secure shredding service. |
